# Exploring the Impact of COVID-19 on Ulcerative Colitis Patients: A Lifestyle Perspective

**DOI:** 10.3390/medicina60010182

**Published:** 2024-01-20

**Authors:** Zane Straume, Nikola Krūmiņa, Ilze Elbere, Maija Rozenberga, Dace Rudzīte, Anna Proskurina, Juliana Ozoliņa, Jānis Kloviņš, Vita Skuja, Angelika Krūmiņa

**Affiliations:** 1 Riga East Clinical University Hospital, Gastroenterology, Hepatology and Nutrition Clinic, Hipokrata Street 2, LV-1038 Riga, Latvia; 000996@rsu.edu.lv (J.O.); vita.skuja@rsu.lv (V.S.); 2 Department of Internal Diseases, Riga Stradins University, Dzirciema Street 16, LV-1007 Riga, Latvia; nikola.krumina@rsu.lv (N.K.); anna.proskr@gmail.com (A.P.); 3 Latvian Biomedical Research and Study Centre, Ratsupites Street 1, LV-1067 Riga, Latvia; ilze.elbere@biomed.lu.lv (I.E.); klovins@biomed.lu.lv (J.K.); 4 Laboratory “Gailezers”Riga East Clinical University Hospital, Hipokrata Street 2, LV-1038 Riga, Latvia; dace.rudzite@aslimnica.lv; 5 Pauls Stradins Clinical University Hospital, Pilsonu Street 13, LV-1002 Riga, Latvia; 6 Department of Infectology, Riga Stradins University, Dzirciema Street 16, LV-1007 Riga, Latvia; angelika.krumina@rsu.lv

**Keywords:** ulcerative colitis, COVID-19, cross-sectional study, lifestyle factors

## Abstract

*Background and Objectives*: Severe acute respiratory syndrome coronavirus 2 (SARS-CoV-2 is the new coronavirus that caused the coronavirus disease 2019 (COVID-19) outbreak. Studies have increasingly reported the involvement of organs outside the respiratory system, including the gastrointestinal tract. Data on the association between COVID-19 and ulcerative colitis (UC) are lacking. *Materials and Methods*: In this one-centre cross-sectional study, 49 patients with UC from the Riga East Clinical University Hospital outpatient clinic were included from June 2021 to December 2021. The patients were divided into two groups according to their history of a confirmed positive or negative COVID-19 status. Data on their lifestyle, diet, and medications and the food supplements used by the patients were collected during interviews and analysed using the R 4.2.1 software. *Results*: Out of 49 patients, 33 (63.3%) were male and 13 (36.7%) were female, with a mean age of 32.33 ± 8.6 years. Fourteen patients (28.6%) had a confirmed COVID-19 infection in the last year. The most common COVID-19-related symptoms were a fever and rhinorrhoea. A third of patients followed the inflammatory bowel disease diet (16; 32.7%); out of these patients, 12 (34.3%) did not contract COVID-19 (OR: 0.78 (0.18; 2.98), *p* > 0.05). In the COVID-19-positive group, the majority of patients did not use vitamin D (11; 79% vs. 3; 21%, (OR: 0.38 (0.07; 1.51), *p* = 0.28) or probiotics (11; 78.6% vs. 3; 21.4%, OR: 1.33 (0.23; 6.28), *p* = 0.7). In the COVID-19-positive group, most patients did not smoke (12; 85.7% vs. 2; 14.3%, *p* = 0.475) and did not use alcohol (9; 64.3% vs. 5; 35.7%, OR: 0.63 (0.16; 2.57), *p* = 0.5). Most of the patients who participated in sports activities were COVID-negative (18; 51.4% vs. 6; 42.9%, *p* = 0.82). *Conclusions*: There were no statistically significant differences in the use of food supplements, probiotics, or vitamins; the lifestyle habits; or the COVID-19 status in patients with UC.

## 1. Introduction

Severe acute respiratory syndrome coronavirus 2 (SARS-CoV-2), first identified in China at the end of 2019, is a beta coronavirus that is transmitted by airway droplets from human to human [1] and that caused the coronavirus disease 2019 (COVID-19) outbreak [2,3]. SARS-CoV-2 expresses surface spike proteins that bind to various receptors of human cells (CD26, CD147, and CD209), with the type 2 angiotensin-converting enzyme (ACE2) as the main target. ACE2 is highly expressed in respiratory and gastrointestinal systems, particularly in the oesophageal epithelium, glandular gastric mucosa, enterocytes, colonocytes [4], and endothelial cells [1]. Most patients have gastrointestinal symptoms, such as nausea, vomiting, anorexia, abdominal pain, and diarrhoea, while some patients only show gastrointestinal symptoms [4,5].

Data on the association between COVID-19 and ulcerative colitis (UC) are limited. The risk factors for a more severe COVID-19 disease in patients with inflammatory bowel disease (IBD) are an active disease and UC—a chronic, idiopathic, immune-mediated inflammatory disorder of the digestive tract [2]. In general, IBD affects nearly 3 million people in the United States and 2.5–3 million people in Europe, with a direct healthcare cost of EUR 4.6–5.6 billion annually in Europe, mostly due to hospitalisations and surgeries [6,7]. The annual incidence of UC is estimated at 5–15 new cases per 100,000 people [8]. The treatment of IBD is aimed at controlling an overactive immune response, which may involve the use of immune-modifying therapies, including immunomodulators or biologic drugs. Many of these treatments are associated with a known increased risk of infection, including an increased risk of a SARS-CoV-2 infection [6,9]. One of the big issues for IBD patients is therapeutic adherence, which has been reduced for COVID-19 with all the potential derivative implications. In addition, the vaccination rates against SARS-CoV-2 are affected by a fear of worsening the course of IBD or experiencing major side effects from the vaccine [10]. The published data suggest that the use of vitamins and dietary supplements also increases in patients exhibiting COVID-related anxiety, with the most popular supplements being vitamin D, vitamin C, and multivitamin products [11]. This study aims to bridge the gap in understanding the specific impact of COVID-19 on patients with UC, particularly focusing on lifestyle factors. 

## 2. Materials and Methods

### 2.1. Study Design and Subjects

In this cross-sectional study, 49 ulcerative colitis patients who attended the outpatient clinic of the Riga East Clinical University Hospital from June 2021 to December 2021 were enrolled. The inclusion criteria were: an age over 18 years, a previous diagnosis of UC, and signed informed consent to participate in the study. Patients with other IBDs (Crohn’s disease or microscopic colitis), other types of colitis, a history of colon cancer, or a history of other malignant tumours with current radiation or immunosuppressive treatment and patients with an intellectual disability were excluded from the study. The time frame was selected to capture the most recent and relevant data following the initial COVID-19 wave. All the patients were divided into two groups according to their history of COVID-19. Patients were assigned to the COVID-19-positive group only if they had a documented positive SARS-CoV-2 test. Data on their lifestyle (stress level, smoking, alcohol consumption, and sports activities), diet, medications, and food supplements used were collected during an interview. Ethical approval for this study was obtained from the Riga East Clinical University Hospital Medical and Biomedical Research Ethical Committee (No. 14/2021). All the participants provided informed consent.

### 2.2. Statistical Analysis

The data were analysed using the R 4.2.1 software (R Core Team (2022), R Foundation for Statistical Computing, Vienna, Austria). Categorical variables were expressed as numbers (*N*) and percentages (%). A binomial test was used to compare two categorical proportions. Independent groups were analysed using Pearson’s chi-squared test (if the expected frequencies were >5) and Fisher’s exact test (if the expected frequencies were <5). An odds ratio (OR) calculation was used to evaluate the 2 × 2 tables. The normality of the distribution of continuous variables was checked using the Shapiro–Wilk test. Normally distributed continuous variables were presented as the mean (M) and standard deviation (SD). Data without a normal distribution were presented as the median (Md) and interquartile range (Q1–Q3). Two independent and normally distributed samples were compared using Student’s independent-sample *t*-test; otherwise, the Mann–Whitney test was used. An analysis of variance (ANOVA) was used for analyses of three or more groups, and a Bonferroni post hoc *t*-test was used when the ANOVA resulted in a *p* < 0.05. Nonparametric data were analysed via Kruskal–Wallis tests. Pearson’s correlation coefficient (denoted r) was used for calculating the correlation of two normally distributed quantitative variables, and otherwise, Spearman’s correlation coefficient (denoted rs) was used. The following classification was used to evaluate the correlation: <0.3—weak; 0.31–0.69—average; and >0.7—strong. A linear regression analysis was performed to assess the relationship between two quantitative variables. A 95% confidence interval (95% CI) was calculated to evaluate the accuracy of the statistical parameters. In all the statistical analyses, a *p*-value < 0.05 was considered statistically significant.

## 3. Results

### 3.1. Patient Demographics and Characteristics

In six months, 49 patients with UC were enrolled in the study. Out of 49 patients, 31 (63%) were male and 18 (37%) were female. The men were taller than the women, with a median height of 178 cm (172–186) vs. 167 cm for women (164–173), and they had a higher median weight—76.0 kg (70.0–88.5) vs. 66.0 kg (58.8–72.0) (Table 1). The median body mass index (BMI) was 26.8 kg (23.9–27.7) in the COVID-19-positive patient group and 22.8 kg (I21.8–28.6) in the COVID-19-negative patient group (OR: 1.04 (0.81; 1.32), *p* = 0.73). 

Among the 31 male patients included in the study, 7 (23%) had a history of COVID-19. Of the 18 female patients, 7 (39%) were COVID-19-positive. There was no statistical significance between the genders with respect to COVID-19 (*p* = 0.73). The mean age in the COVID-19-positive group was 38 years (33.3; 44.8), and in the COVID-19-negative group, it was 38.0 years (36.0; 53.5); there was no statistical significance (*p* = 0.36). The median duration of COVID-19 symptoms was 7.0 days [3.50; 18.8]. 

In COVID-19-positive patients, the most common symptoms (Table 2) were a fever (11; 78.6% vs. 3; 21.4%, *p* < 0.001) and rhinorrhoea (6; 42.9% vs. 8; 57.1%, *p* < 0.001). Only three patients reported diarrhoea (3; 21.4% vs. 11; 78.6%, *p* = 0.002).

### 3.2. Diet and COVID-19

More than half of the patients (25; 51%) followed a specific diet, with the most common being the anti-inflammatory, or IBD, diet (16 patients; 32.7%, *p* < 0.05). Other specific diets included the lactose-free diet (two patients; 4.08%, *p* < 0.001) and the diabetes diet (two patients; 4.08%, *p* < 0.001). Out of all the patients who followed the IBD diet, 12 (34.3%) were COVID-19-negative and 4 (28.6%) were COVID-19-positive (OR: 0.78 (0.18; 2.98), *p* > 0.05) (Table 3).

### 3.3. Use of IBD-Specific Medications

Most patients (71.4%) received mesalazine as a pathogenetic treatment for IBD. In the COVID-19-negative group, 25 (71.4%) patients used mesalazine, compared to 10 (28.6%) patients in the COVID-19-positive group (Table 4).

### 3.4. Use of Probiotics

In both the positive and negative COVID-19 groups, most of the patients did not use any probiotics in the month before the interview. Only three patients used probiotic supplements at the time of the interview; of those, one (7.14%) was positive for COVID-19 and two (5.71%) were negative for COVID-19 (OR: 1.34 (0.04; 17.8), *p* > 0.05) (Table 5).

### 3.5. Vitamin and Food Supplement Intake 

Of the 49 patients, 25 (44.6%) used different food supplements: 9 (18.4%) patients used magnesium, 5 (10.2%) used fish oil, and 4 (8.16%) used iron. Of these patients, 17 (48.6%) were COVID-19-negative and 8 (57.1%) were COVID-19-positive (*p* = 0.821) (Table 6). In general, women used food supplements more than men (14; 77.8% vs. 11; 35.5%) (OR: 5.98 (1.66; 26.3), *p* = 0.01). 

Of the 49 patients, 18 (36.7%) used supplementary vitamin D. In the COVID-19-negative group, 15 (43%) patients used vitamin D, while in the COVID-19-positive group, only 3 (21%) patients used it.

**Figure 1:**
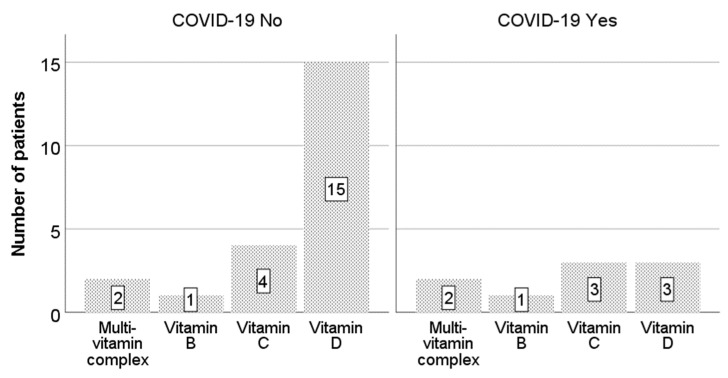
Use of vitamins according to COVID-19 group.

Vitamin C was less commonly used—by seven patients (14.3%). In the COVID-19-positive group, three patients (21%) used vitamin C, while in the COVID-19-negative group, four patients (11%) used it. Other products used included multivitamin complexes and B vitamins (Figure 1).

### 3.6. Lifestyle Habits and COVID-19

#### 3.6.1. Smoking

In the COVID-19-positive group, two patients (14.3%) were smokers. In the COVID-19-negative group, there were 9 smokers (25.7%) and 26 non-smokers (74.3%) (OR: 0.51 (0.06; 2.45), *p* = 0.47) (Table 7).

#### 3.6.2. Alcohol Consumption

Most of the patients consumed some type of alcohol (35; 71.4% alcohol users vs. 14; 28.6% non-users, *p* < 0.05). Most of the used alcoholic beverages were wine (15; 30.6%), vodka (14; 28.6%), or beer (9; 18.4%). Most patients drank alcohol once a month (22; 44.9%) or one to two times a week (13; 26.5%).

Alcohol users made up 64.3% of patients in the COVID-19-positive group and 74.3% in the COVID-19-negative group (OR: 0.63 (0.16; 2.57), *p* = 0.5). In the COVID-19-negative group, 15 (42.9%) patients drank alcoholic beverages once a month, compared to 7 (50%) in the COVID-19-positive group (*p* = 0.89). There were 11 (31.4%) patients in the COVID-19-negative group and 2 (14.3%) patients in the COVID-19-positive group who consumed alcohol 1–2 times per week (*p* = 0.29).

#### 3.6.3. Stress Level

Eighteen patients reported feeling high stress in the last month: twelve (34.3%) in the COVID-19-negative group and six (42.9%) in the COVID-19-positive group (OR: 1.43 (0.38; 5.22), *p* = 0.81). More patients in the COVID-19-negative group had lower self-reported stress levels than in the COVID-19-positive group (23; 65.7% vs. 12; 34.3%).

#### 3.6.4. Sports Activities

Of the 49 patients, 24 (49%) engaged in sports activities, most commonly (13; 26.5%) at least three times a week. Five patients (10.2%) were physically active daily. The most common activities were running, cycling, and swimming, each performed by eight patients (16.3%) (*p* < 0.001). Of the 24 physically active patients, most were negative for COVID-19 (18; 75% vs. 6; 25%) (Table 8).

## 4. Discussion

In this cross-sectional study, we analysed the different characteristics of outpatients with UC—anthropometric data, the use of IBD medications and dietary supplements, and lifestyle habits and UC treatment—and their possible association with SARS-CoV-2 infections. We also evaluated the symptoms and severity of COVID-19 infections in patients who were positive for SARS-CoV-2.

Regarding the anthropometric data, reports in the literature show patients with UC being slightly shorter and leaner (with a smaller BMI) than healthy controls [12,13], with a median BMI in the healthy weight range [12,14]. Male patients with UC were taller than females and had a higher weight. Compared to other studies, the differences in height in late adolescence were not statistically significant compared to healthy controls [12]. In this study, patients in the COVID-19-positive group had a higher BMI than those in the COVID-19-negative group. There were no overweight patients with UC in our study. The age of UC onset was similar to that reported in the literature, predominantly between 30 and 40 years of age in adult patients [7,12]. 

The mean duration of COVID-19 symptoms was seven days in our study. The clinical spectrum of COVID-19 is variable. Epidemiological studies have reported that the vast majority (80%) of patients infected with SARS-CoV-2 are asymptomatic or show mild symptoms; approximately 20% progress to a severe disease, of which 5% develop acute respiratory distress syndrome, septic shock, or multi-organ failure with a high mortality risk. The most frequent symptoms are a fever (up to 50–60%), a cough (40%), and anosmia/ageusia (40%). Diarrhoea is seen in approximately 18% of patients [15]. The natural history of COVID-19 and the evolution of the SARS-CoV-2 infection were conditioned by cell tropism and the host immune response [16,17]. In our study, no patients had severe disease and the most common symptoms related to COVID-19 were similar to those reported in the literature [18,19,20]. 

Depending on the extent and severity of IBD, patients may receive treatment with 5-ASA, biologics, corticosteroids, thiopurines, or molecular-targeting agents. One of the most widely used drug classes is 5-ASA, which corresponds to the findings in our study [21,22,23,24]. Reports have shown that a SARS-CoV-2 infection may contribute to UC flares [25,26].

More than half of the patients in this study (51%) followed specific diets, most commonly the anti-inflammatory IBD diet. Most patients on the IBD diet were COVID-19-negative, though the result was not statistically significant, likely due to the small sample size. We did not find any studies on diet and COVID-19 in patients with IBD. Experts from the European Society for Clinical Nutrition and Metabolism (ESPEN) state that, beyond the dietary management of IBD, nutritional optimisation and the treatment of both malnutrition and obesity-related illnesses are important to best equip patients to face COVID-19 [27]. Preserving the nutritional status and preventing or treating malnutrition have the potential to reduce complications and negative outcomes. As COVID-19 can be accompanied by nausea, vomiting, and diarrhoea, impairing food intake and absorption, a good nutritional status is an advantage for people at risk of severe COVID-19 [27,28].

Almost half of our patients used different kinds of food supplements, mostly magnesium, fish oil, and iron. Women used food supplements more often than men. In a cross-sectional study conducted in Greece to evaluate the use of dietary supplements and their association with other factors, particularly anxiety related to COVID-19, it was found that 62.6% of the participants used dietary supplements. The most popular supplements were vitamin D, followed by vitamin C, multivitamins, and mineral products. The researchers found that women, former smokers, and people exhibiting signs of COVID-19-related anxiety were approximately two times more likely to use dietary supplements of any kind [11]. While it is important to prevent and treat micronutrient deficiencies, there is no established evidence that the routine, empirical use of a supraphysiological or supratherapeutic amount of micronutrients can prevent or improve the clinical outcomes in COVID-19 [27]. In a prospective, randomised, double-blind, placebo-controlled, multicentre trial, oral zinc administration in COVID-19 patients reduced the 30-day mortality, the chance of admission to the intensive care unit, and the duration of the symptoms [29]. In another randomised clinical trial of outpatients with COVID-19, high-dose zinc gluconate, ascorbic acid, or a combination of the two supplements did not significantly decrease the duration of the symptoms compared to the standard of care [30].

Most of the patients in our study, in both the COVID-19-positive and the COVID-19-negative groups, did not use probiotics. As millions of people have been infected during the ongoing COVID-19 pandemic, the performed studies strongly suggest that gut microbiota modulation could facilitate a timely recovery from COVID-19 and reduce the risk of post-acute COVID-19 syndrome (PACS) [31]. A variety of possible microbiota-based prophylaxes and therapies for COVID-19, including faecal microbiota transplantation, probiotics, and prebiotics, have been discussed. The China National Health Commission has recommended the administration of probiotics to patients with severe COVID-19 to restore and maintain the intestinal microflora balance and prevent secondary infections [31]. Supplementation with microbiota-targeted substrates (prebiotics), such as specific dietary fibres and/or the direct transfer of one or several specific beneficial microbiota (probiotics), are promising approaches to COVID-19 treatment that modulate the gut microbiota [32,33]. For example, a Lactococcus lactis strain was engineered to express and secrete the anti-inflammatory cytokine IL-10 to treat colitis [34].

In the COVID-19-positive group, most of the patients did not use vitamin D. In the COVID-19-negative group, more patients supplemented with vitamin D, but in both cases, the results were not statistically significant. We collected data on vitamin intake, but not the precise dosage. Active vitamin D (1,25(OH)2D3—calcitriol) exerts multiple biological properties (endocrine, paracrine, and intracrine) in the human body [35]. The vitamin D receptor is almost ubiquitously expressed by the cells of the immune system, supporting the role of vitamin D in the regulation of acute and chronic inflammatory responses. Recently, a robust meta-analysis of more than 1500 articles on this topic identified vitamin D supplementation as a protective factor against acute airway infections, thanks to its immunomodulatory properties [1,36]. Low serum concentrations of 25(OH)D3, especially below 25 nmol/L, are a risk factor for susceptibility to viral respiratory infections [36]. Various studies with vitamin D supplementation in COVID-19 patients have shown an accelerated recovery, a reduction in the need for intensive care, and a reduction in mortality with oral calcifediol and cholecalciferol [37,38]. Vitamin D supplementation seems to be effective in COVID-19 when administered for a medium or long term, while high and/or single doses were found to be ineffective [1].

Vitamin C was used by 14% of the UC patients in our study. The antioxidant properties of vitamin C have led to a hypothesis about its neuroprotective properties [39]. It is difficult to find unambiguous results that indicate such an effect in vivo [40]. Stimulating the immune system with high doses of vitamin C may paradoxically reduce immunity [41]. A meta-analysis showed that the use of vitamin C in randomised trials was associated with a significant reduction in the in-hospital mortality for patients with COVID-19, compared to patients who did not receive vitamin C [42]. In one study, the use of vitamin C with other supplements (including zinc and vitamin D) did not reduce the in-hospital mortality, possibly due to multicomponent supplementation being used in patients with more severe disease [43].

In general, low levels of vitamins A, E, B6, B12, zinc, and selenium have been associated with adverse clinical outcomes during viral infections. An assessment of vitamin levels, the intake of Ω-3 polyunsaturated fatty acids, and the selenium, zinc, and iron levels should be considered in patients with COVID-19 [27,44]. We suggest that the provision of daily allowances for vitamins and trace elements be ensured for malnourished patients at risk for or with COVID-19, aiming to maximise the general nutritional defence against infection [27].

In 2020, 22.3% of the world’s population used tobacco: 36.7% of men and 7.8% of women [45]. Smoking is associated with an increased expression of angiotensin-converting enzyme (ACE), which is the main receptor that allows the penetration of SARS-CoV-2 into human cells [18]. In our study, there were more non-smokers in the COVID-19-negative group, though the results were not statistically significant. Studies have shown a negative association between smoking and COVID-19 infections [46,47]. Current smokers have an excess risk of 34% for developing a severe course of COVID-19 (based on 124 studies) and an excess risk of 32% for mortality (based on 119 studies) [48]. 

The results of a large study that evaluated alcohol use during the COVID-19 pandemic in the United Kingdom suggested that being older is associated with increased alcohol consumption [49]. In our study, the COVID-19-positive group had more alcohol users, though this was not statistically significant. Although many studies have emphasised the increase in the consumption of alcoholic beverages during the pandemic [50,51,52,53], most of our study participants did not use alcohol as often, mostly 1–2 times a week.

In the COVID-19-positive group, only a few patients self-reported high stress levels in the last month before the interview; most people in both groups admitted less stress in the last month. In some studies on the stress levels in patients with IBD during the COVID-19 pandemic, the patients showed depression, anxiety, and fear associated with COVID-19, including a fear of being diagnosed with the infection [54,55,56]. Furthermore, the lockdowns imposed by COVID-19 had a negative effect on the lifestyle and psychological stress of patients with IBD [22]. Half of our study patients remained physically active by participating in sports activities. Most participants engaged in physical exercise 1–3 times a week. Patients who were physically active were less likely to contract COVID-19, although without statistical significance. In a study by Yu et al., one of the self-reported factors that influenced IBD symptoms in patients during the COVID-19 pandemic was a “reduction of exercise”. The results showed that the decrease in physical activity was a risk factor for a worsening disease [23], although the relationship between exercise and IBD activity is not clear. Another study showed that the proportion of patients who were stressed due to COVID-19 rendering them unable to exercise and who stayed indoors increased significantly during lockdown [57]. In a 2023 study of physical activity in IBD patients, almost half (42%) of the participants were not sufficiently active. Furthermore, most of the physically inactive patients believed that physical exercise might worsen their IBD symptoms [14]. The advice of ESPEN experts to increase physical activity during lockdown included walking in the house and to the store, lifting and carrying groceries, alternating leg lunges, climbing stairs, and performing stand-to-sit and sit-to-stand exercises, chair squats, sit-ups, and push-ups. The use of eHealth and exercise videos, which focus on encouraging and delivering physical activity through the Internet, mobile technologies, and television, are other options to maintain physical function and mental health. Everyday physical activity for more than 30 min (or every other day for more than 1 h) is recommended to maintain fitness, mental health, muscle mass, and thus, energy expenditure and body composition [27].

## 5. Conclusions

This cross-sectional study investigated the lifestyle habits, diet, medication, and supplement use in ulcerative colitis patients and the possible influence of these factors on COVID-19 morbidity. There was no statistical significance in the positive and negative COVID-19 groups with respect to IBD treatment; the use of dietary supplements, probiotics, or vitamins; or lifestyle factors such as smoking, alcohol consumption, and stress levels. Patients with UC need careful follow-up and evaluations of the course of their IBD, as COVID-19 can cause flare-ups as well as induce the new onset of ulcerative colitis. Patients with UC should be encouraged to get vaccinated against SARS-CoV-2 [58]. Importantly, SARS-CoV-2 vaccines have shown a good safety profile in IBD patients on biologic therapy [59,60]. Patients should also be informed about the possible benefits of supplementation with vitamin D. 

The limitations of this study include its small sample size and single-centre design. However, this study provides valuable insights into the factors potentially affecting COVID-19 in UC patients and contributes to a better understanding of the disease’s systemic impacts, as the available literature on UC patients in this context is scarce. With the COVID-19 pandemic ongoing, this study’s results can be used to guide the recommendations for UC patients. 

## Figures and Tables

**Table 1:** Characteristics in ulcerative colitis patients.

Characteristic Feature	Male	Female	OR (95% CI)	*p*-Value
	*N* = 31	*N* = 18		
Age, years, Md [Q1; Q3]	38.0 [34.5; 49.0]	39.0 [34.5; 50.2]	0.99 [0.94; 1.04]	0.94
Height, cm, Md [Q1; Q3]	178 [172; 186]	167 [164; 173]	0.82 [0.72; 0.92]	<0.001
Weight, kg, Md [Q1; Q3]	76.0 [70.0; 88.5]	66.0 [58.8; 72.0]	0.95 [0.90; 1.00]	0.011
BMI, kg/m^2^, Md [Q1; Q3]	24.8 [21.8; 28.8]	22.5 [22.0; 26.8]	0.97 [0.79; 1.19]	0.58
Age at first manifestation of UC, years, Md [Q1; Q3]	31.0 [24.0; 40.5]	31.5 [24.2; 38.8]	0.98 [0.93; 1.03]	0.79
Age at diagnosis of UC, years, Md [Q1; Q3]	31.0 [26.5; 40.5]	31.5 [27.2; 38.8]	0.98 [0.93; 1.03]	0.74
Total number of UC exacerbations, Md [Q1; Q3]	4 [2; 13]	2 [1; 9]	0.99 [0.94; 1.04]	0.12
Number of UC exacerbations, last year, Md [Q1; Q3]	1 [0; 1]	1 [0; 1]	0.94 [0.53; 1.66]	0.9

**Table 2:** Complaints according to COVID-19 status.

	COVID-19-Negative	COVID-19-Positive	OR (95% CI)	*p*-Value
COVID-19 symptoms, *N* (%):				<0.001
	35 (100%)	0 (0.00%)	Ref.	
No	0 (0.00%)	2 (14.3%)		
Yes	0 (0.00%)	12 (85.7%)		
Rhinorrhoea, *N* (%):				<0.001
No	35 (100%)	8 (57.1%)	Ref.	
Yes	0 (0.00%)	6 (42.9%)		
Nasal congestion, *N* (%):				0.001
No	35 (100%)	9 (64.3%)	Ref.	
Yes	0 (0.00%)	5 (35.7%)		
Dysosmia, *N* (%):				0.001
No	35 (100%)	9 (64.3%)	Ref.	
Yes	0 (0.00%)	5 (35.7%)		
Dysgeusia, *N* (%):				0.28
No	35 (100%)	13 (92.9%)	Ref.	
Yes	0 (0.00%)	1 (7.14%)		
Sore throat, *N* (%):				0.005
No	35 (100%)	10 (71.4%)	Ref.	
Yes	0 (0.00%)	4 (28.6%)		
Cough, *N* (%):				0.005
No	35 (100%)	10 (71.4%)	Ref.	
Yes	0 (0.00%)	4 (28.6%)		
Chest discomfort, *N* (%):				0.077
No	35 (100%)	12 (85.7%)	Ref.	
Yes	0 (0.00%)	2 (14.3%)		
Tachycardia, *N* (%):				0.28
No	35 (100%)	13 (92.9%)	Ref.	
Yes	0 (0.00%)	1 (7.14%)		
Nausea, *N* (%):				0.2
No	35 (100%)	13 (92.9%)	Ref.	
Yes	0 (0.00%)	1 (7.14%)		
Vomiting, *N* (%):				0.28
No	35 (100%)	13 (92.9%)	Ref.	
Yes	0 (0.00%)	1 (7.14%)		
Abdominal pain, *N* (%):				0.286
No	35 (100%)	13 (92.9%)	Ref.	
Yes	0 (0.00%)	1 (7.14%)		
Bloating, *N* (%):				
No	35 (100%)	14 (100%)	Ref.	
Diarrhoea, *N* (%):				0.02
No	35 (100%)	11 (78.6%)	Ref.	
Yes	0 (0.00%)	3 (21.4%)		
Constipation, *N* (%):				0.286
No	35 (100%)	13 (92.9%)	Ref.	
Yes	0 (0.00%)	1 (7.14%)		
Loss of appetite, *N* (%):				0.28
No	35 (100%)	13 (92.9%)	Ref.	
Yes	0 (0.00%)	1 (7.14%)		
Postprandial fullness, *N* (%):				0.28
No	35 (100%)	13 (92.9%)	Ref.	
Yes	0 (0.00%)	1 (7.14%)		
Headache, *N* (%):				0.005
No	35 (100%)	10 (71.4%)	Ref.	
Yes	0 (0.00%)	4 (28.6%)		
Dizziness, *N* (%):				0.28
No	35 (100%)	13 (92.9%)	Ref.	
Yes	0 (0.00%)	1 (7.14%)		
Bone pain, *N* (%):				0.07
No	35 (100%)	12 (85.7%)	Ref.	
Yes	0 (0.00%)	2 (14.3%)		
Fever, *N* (%):				<0.001
No	35 (100%)	3 (21.4%)	Ref.	
Yes	0 (0.00%)	11 (78.6%)		
Weakness, *N* (%):				0.001
No	35 (100%)	9 (64.3%)	Ref.	
Yes	0 (0.00%)	5 (35.7%)		
Confusion, *N* (%):				0.28
No	35 (100%)	13 (92.9%)	Ref.	
Yes	0 (0.00%)	1 (7.14%)		

**Table 3:** Diets followed by UC patients and their COVID-19 status.

Diet	COVID-19-Negative	COVID-19-Positive	OR (95% CI)	*p*-Value
Followed a specific diet, *N* (%):				0.82
No	18 (51.4%)	6 (42.9%)	Ref.	
Yes	17 (48.6%)	8 (57.1%)	1.40 [0.39; 5.18]	
IBD diet, *N* (%):				0.99
No	23 (65.7%)	10 (71.4%)	Ref.	
Yes	12 (34.3%)	4 (28.6%)	0.78 [0.18; 2.98]	
Intermittent fasting, *N* (%):				0.99
No	34 (97.1%)	14 (100%)	Ref.	
Yes	1 (2.86%)	0	. [.; .]	
Vegan, *N* (%):				0.99
No	34 (97.1%)	14 (100%)	Ref.	
Yes	1 (2.86%)	0 (0.00%)	. [.; .]	
Vegetarian, *N* (%):				0.99
No	34 (97.1%)	14 (100%)	Ref.	
Yes	1 (2.86%)	0 (0.00%)		
Lactose-free, *N* (%):				0.503
No	33 (97.1%)	13 (92.9%)	Ref.	
Yes	1 (2.94%)	1 (7.14%)	2.48 [0.06; 102]	
Gluten-free, *N* (%):				0.28
No	35 (100%)	13 (92.9%)	Ref.	
Yes	0	1 (7.14%)		
Low-FODMAP * diet, *N* (%):				0.28
No	35 (100%)	13 (92.9%)	Ref.	
Yes	0	1 (7.14%)		
Diabetes diet, *N* (%):				0.07
No	35 (100%)	12 (85.7%)	Ref	
Yes	0	2 (14.3%)		

* FODMAP—fermentable oligosaccharides, disaccharides, monosaccharides, and polyols.

**Table 4:** Treatment of ulcerative colitis according to COVID-19 group.

Used Medications	COVID-19-Negative	COVID-19-Positive	OR (95% CI)	*p*-Value
Sulfasalazine, *N* (%)				0.99
No	31 (88.6%)	13 (92.9%)	Ref.	
Yes	4 (11.4%)	1 (7.14%)	0.66 [0.02; 5.34]	
Mesalazine, *N* (%):				0.99
No	10 (28.6%)	4 (28.6%)	Ref.	
Yes	25 (71.4%)	10 (71.4%)	0.99 [0.25; 4.44]	
Azathioprine, *N* (%):				0.39
No	31 (88.6%)	11 (78.6%)	Ref.	
Yes	4 (11.4%)	3 (21.4%)	2.10 [0.34; 11.7]	
Biological, *N* (%):				0.53
No	31 (88.6%)	12 (85.7%)	Ref.	
Yes	3 (8.57%)	1 (7.14%)		
Adalimumab	1 (2.86%)	0 (0.00%)		
Infliximab	0 (0.00%)	1 (7.14%)		
Other medications, *N* (%):				0.32
None	33 (94.3%)	12 (85.7%)	Ref.	
Antidepressants	1 (2.86%)	1 (7.14%)		
1, 2	0 (0.00%)	1 (7.14%)		
Pancreatin	1 (2.86%)	0 (0.00%)		

**Table 5:** Use of probiotics according to COVID-19 group.

	COVID-19-Negative	COVID-19-Positive	OR (95% CI)	*p*-Value
Probiotics at the time of interview, N (%):				0.99
No	33 (94.3%)	13 (92.9%)	Ref.	
Yes	2 (5.71%)	1 (7.14%)	1.34 [0.04; 17.8]	
Probiotics last month, N (%):				0.7
No	29 (82.9%)	11 (78.6%)	Ref.	
Yes	6 (17.1%)	3 (21.4%)	1.33 [0.23; 6.28]	
Probiotic type				
Probiotic consisting of: *Bifidobacterium infantis 35624*^®^ bacteria—1 × 10 (9) CFUs	1 (3.23%)	0 (0.00%)		
Probiotic consisting of: *Saccharomyces boulardii*	3 (9.68%)	1 (5.56%)		
Probiotic consisting of: *Lactobacillus acidophilus*, *Lactobacillus casei*, *Lactobacillus plantarum*, *Lactobacillus reuteri*, *Lactobacillus rhamnosus*, *Bifidobacterium longum*, and *Streptococcus thermophilus*	0 (0.00%)	1 (5.56%)		
Probiotic consisting of: *Enterococcus faecium*, *Lactobacillus acidophilus*, and *Bifidobacterium infantis*	2 (6.45%)	0 (0.00%)		
Probiotic consisting of: *Streptococcus thermophiles*, *Bifidobacterium* (*B. breve*, *B. longum* *, and *B. infantis* **), and *laktobacilli* (*L. acidophilus*, *L. plantarum*, *L. paracasei*, and *L. delbrueckii subsp. bulgaricus*)	1 (3.23%)	0 (0.00%)		

* Reclassified as *B. lactis*; ** Reclassified as *L. helveticus*.

**Table 6:** Use of food supplements according to COVID-19 group.

	COVID-19-Negative	COVID-19-Positive	OR (95% CI)	*p*-Value
Food supplements used last month, *N* (%):				0.82
No	18 (51.4%)	6 (42.9%)	Ref.	
Yes	17 (48.6%)	8 (57.1%)	1.40 [0.39; 5.18]	
Fish oil, *N* (%):				0.99
No	31 (88.6%)	13 (92.9%)	Ref.	
Yes	4 (11.4%)	1 (7.14%)	0.66 [0.02; 5.34]	
Evening primrose oil, *N* (%):				0.49
No	34 (97.1%)	13 (92.9%)	Ref.	
Yes	1 (2.86%)	1 (7.14%)	2.56 [0.06; 105]	
Spirulina, *N* (%):				0.07
No	35 (100%)	12 (85.7%)	Ref.	
Yes	0 (0.00%)	2 (14.3%)		
Protein powder, *N* (%):				0.99
No	34 (97.1%)	14 (100%)	Ref.	
Yes	0 (0.00%)	1 (7.14%)		
Curcumin, *N* (%):				0.99
No	34 (97.1%)	14 (100%)	Ref.	
Yes	1 (2.86%)	0 (0.00%)		
Collagen, *N* (%):				0.99
No	34 (97.1%)	14 (100%)	Ref.	
Yes	1 (2.86%)	0 (0.00%)		
Red yeast rice extract, *N* (%):				0.99
No	34 (97.1%)	14 (100%)	Ref.	
Yes	1 (2.86%)	0 (0.00%)		
Boswellia, *N* (%):				0.99
No	34 (97.1%)	14 (100%)	Ref.	
Yes	1 (2.86%)	0 (0.00%)		
Copper, *N* (%):				0.99
No	34 (97.1%)	14 (100%)	Ref.	
Yes	1 (2.86%)	0 (0.00%)		
Selenium, *N* (%):				0.99
No	34 (97.1%)	14 (100%)	Ref.	
Yes	1 (2.86%)	0 (0.00%)		
Iron, *N* (%):				0.06
No	34 (97.1%)	11 (78.6%)	Ref.	
Yes	1 (2.86%)	3 (21.4%)	8.13 [0.85; 250]	
Calcium, *N* (%):				0.49
No	34 (97.1%)	13 (92.9%)	Ref.	
Yes	1 (2.86%)	1 (7.14%)	2.56 [0.06; 105]	
Magnesium, *N* (%):				0.35
No	28 (80.0%)	12 (85.7%)	Ref.	
Yes	7 (20.0%)	2 (14.3%)	0.70 [0.08; 3.56]	
Zinc, *N* (%):				0.99
No	33 (94.3%)	14 (100%)	Ref.	
Yes	2 (5.71%)	0 (0.00%)		

**Table 7:** Smoking habits in UC patients.

	COVID-19-Negative	COVID-19-Positive	OR (95% CI)	*p*-Value
Current smokers, *N* (%):				0.47
No	26 (74.3%)	12 (85.7%)	Ref.	
Yes	9 (25.7%)	2 (14.3%)	0.51 [0.06; 2.45]	
Ex-smokers, *N* (%):				0.99
No	14 (40.0%)	5 (35.7%)	Ref.	
Yes	21 (60.0%)	9 (64.3%)	1.19 [0.33; 4.69]	
Years of smoking, Md [Q1; Q3]	8.00 [5.00; 20.0]	12.5 [3.25; 23.8]	1.02 [0.96; 1.08]	0.98
Cigarettes per day, Md [Q1; Q3]	15.0 [5.50; 20.0]	10.0 [5.00; 12.0]	0.96 [0.87; 1.06]	0.52
Pack years, Md [Q1; Q3]	3.60 [0.00; 8.00]	2.50 [0.00; 8.50]	1.01 [0.95; 1.06]	0.98

**Table 8:** Frequency of sports activities in COVID-19 groups.

	COVID-19-Negative	COVID-19-Positive	OR (95% CI)	*p*-Value
Sports, *N* (%):				0.82
No	17 (68.0%)	8 (32.0%)	Ref.	
Yes	18 (75.0%)	6 (25.0%)	0.72 [0.19; 2.54]	
Once a month, *N* (%):				0.39
No	31 (100%)	18 (100%)	Ref.	
1–2×/week, *N* (%):				
No	28 (90.3%)	14 (77.8%)	Ref.	
Yes	3 (9.68%)	4 (22.2%)	2.58 [0.48; 15.7]	
At least 3×/week, *N* (%):				0.32
No	21 (67.7%)	15 (83.3%)	Ref.	
Yes	10 (32.3%)	3 (16.7%)	0.44 [0.08; 1.77]	
Every day, *N* (%):				0.99
No	28 (90.3%)	16 (88.9%)	Ref.	
Yes	3 (9.68%)	2 (11.1%)	1.19 [0.13; 8.60]	

## Data Availability

The data presented in this study are available from the corresponding author upon request.

## References

[1] Gotelli E., Soldano S., Hysa E., Paolino S., Campitiello R., Pizzorni C., Sulli A., Smith V., Cutolo M. (2022). Vitamin D and COVID-19: Narrative Review after 3 Years of Pandemic. Nutrients.

[2] Bezzio C., Vernero M., Costa S., Armuzzi A., Fiorino G., Ardizzone S., Roselli J., Carparelli S., Orlando A., Caprioli F.A. (2023). SARS-CoV-2 Infection in Patients with Inflammatory Bowel Disease: Comparison between the First and Second Pandemic Waves. BMC Gastroenterol..

[3] Lai C.-C., Shih T.-P., Ko W.-C., Tang H.-J., Hsueh P.-R. (2020). Severe Acute Respiratory Syndrome Coronavirus 2 (SARS-CoV-2) and Coronavirus Disease-2019 (COVID-19): The Epidemic and the Challenges. Int. J. Antimicrob. Agents.

[4] Delgado-Gonzalez P., Gonzalez-Villarreal C.A., Roacho-Perez J.A., Quiroz-Reyes A.G., Islas J.F., Delgado-Gallegos J.L., Arellanos-Soto D., Galan-Huerta K.A., Garza-Treviño E.N. (2021). Inflammatory Effect on the Gastrointestinal System Associated with COVID-19. World J. Gastroenterol..

[5] Xu J., Wu Z., Zhang M., Liu S., Zhou L., Yang C., Liu C. (2021). The Role of the Gastrointestinal System in Neuroinvasion by SARS-CoV-2. Front. Neurosci..

[6] Tripathi K., Godoy Brewer G., Thu Nguyen M., Singh Y., Saleh Ismail M., Sauk J.S., Parian A.M., Limketkai B.N. (2022). COVID-19 and Outcomes in Patients With Inflammatory Bowel Disease: Systematic Review and Meta-Analysis. Inflamm. Bowel Dis..

[7] Barreiro-de Acosta M., Molero A., Artime E., Díaz-Cerezo S., Lizán L., de Paz H.D., Martín-Arranz M.D. (2023). Epidemiological, Clinical, Patient-Reported and Economic Burden of Inflammatory Bowel Disease (Ulcerative Colitis and Crohn’s Disease) in Spain: A Systematic Review. Adv. Ther..

[8] Mounsif S., Setouani H., Nadi A., Ghalim F., Delsa H. (2022). Management of Ulcerative Colitis Flare-Ups in the Era of COVID-19. Cureus.

[9] Summa K.C., Hanauer S.B. (2023). COVID-19 and Inflammatory Bowel Disease. Gastroenterol. Clin. North. Am..

[10] Pellegrino R., Pellino G., Selvaggi F., Federico A., Romano M., Gravina A.G. (2022). Therapeutic Adherence Recorded in the Outpatient Follow-up of Inflammatory Bowel Diseases in a Referral Center: Damages of COVID-19. Dig. Liver Dis..

[11] Marakis G., Kontopoulou L., Konstantinidis G., Papathanasiou I.V., Karpetas G., Mirkopoulou D., Walker A.F., Vasara E. (2023). The Use of Dietary Supplements and Their Association with COVID-19-Related Anxiety among Non-Institutionalized Elderly in Northern Greece. J. Diet. Suppl..

[12] Ghersin I., Khateeb N., Katz L.H., Daher S., Shamir R., Assa A. (2019). Anthropometric Measures in Adolescents With Inflammatory Bowel Disease: A Population-Based Study. Inflamm. Bowel Dis..

[13] Abduljabbar M., Alghamdi R., Althobaiti K., Althubaiti S., Alharthi N., Alosaimi G., Qunq M., Saleh L., Alosaimi M. (2022). The Length of Hospital Stays and Clinical and Therapeutic Characteristics of Patients with COVID-19 Early in the Pandemic in Taif City, KSA: A Retrospective Study. Medicine.

[14] Gravina A.G., Pellegrino R., Durante T., Palladino G., D’Onofrio R., Mammone S., Arboretto G., Auletta S., Imperio G., Ventura A. (2023). Inflammatory Bowel Diseases Patients Suffer from Significant Low Levels and Barriers to Physical Activity: The “BE-FIT-IBD” Study. World J. Gastroenterol..

[15] Ario A.R., Mirembe B.B., Biribawa C., Bulage L., Kadobera D., Wamala R. (2021). Timing of Onset of Symptom for COVID-19 from Publicly Reported Confirmed Cases in Uganda. Pan Afr. Med. J..

[16] Macaluso F.S., Giuliano A., Fries W., Viola A., Abbruzzese A., Cappello M., Giuffrida E., Carrozza L., Privitera A.C., Magnano A. (2023). Severe Activity of Inflammatory Bowel Disease Is a Risk Factor for Severe COVID-19. Inflamm. Bowel Dis..

[17] Guan W., Ni Z., Hu Y., Liang W., Ou C., He J., Liu L., Shan H., Lei C., Hui D.S.C. (2020). Clinical Characteristics of Coronavirus Disease 2019 in China. N. Engl. J. Med..

[18] Zhou F., Yu T., Du R., Fan G., Liu Y., Liu Z., Xiang J., Wang Y., Song B., Gu X. (2020). Clinical Course and Risk Factors for Mortality of Adult Inpatients with COVID-19 in Wuhan, China: A Retrospective Cohort Study. Lancet.

[19] Rubin D.T., Feuerstein J.D., Wang A.Y., Cohen R.D. (2020). AGA Clinical Practice Update on Management of Inflammatory Bowel Disease During the COVID-19 Pandemic: Expert Commentary. Gastroenterology.

[20] Mudgal S.K., Gaur R., Rulaniya S., Latha T., Agarwal R., Kumar S., Varshney S., Sharma S., Bhattacharya S., Kalyani V. (2023). Pooled Prevalence of Long COVID-19 Symptoms at 12 Months and Above Follow-Up Period: A Systematic Review and Meta-Analysis. Cureus.

[21] Richter V., Bermont A., Cohen D.L., Broide E., Shirin H. (2022). Effect of Inflammatory Bowel Disease and Related Medications on COVID-19 Incidence, Disease Severity, and Outcome: The Israeli Experience. Eur. J. Gastroenterol. Hepatol..

[22] Grunert P.C., Reuken P.A., Stallhofer J., Teich N., Stallmach A. (2020). Inflammatory Bowel Disease in the COVID-19 Pandemic: The Patients’ Perspective. J. Crohns Colitis.

[23] Yu M., Ye Z., Chen Y., Qin T., Kou J., Tian D., Xiao F. (2020). Questionnaire Assessment Helps the Self-Management of Patients with Inflammatory Bowel Disease during the Outbreak of Coronavirus Disease 2019. Aging.

[24] Lee M.H., Li H.J., Wasuwanich P., Kim S.E., Kim J.Y., Jeong G.H., Park S., Yang J.W., Kim M.S., Yon D.K. (2023). COVID-19 Susceptibility and Clinical Outcomes in Inflammatory Bowel Disease: An Updated Systematic Review and Meta-Analysis. Rev. Med. Virol..

[25] Nakase H., Hayashi Y., Hirayama D., Matsumoto T., Matsuura M., Iijima H., Matsuoka K., Ohmiya N., Ishihara S., Hirai F. (2022). Interim Analysis of a Multicenter Registry Study of COVID-19 Patients with Inflammatory Bowel Disease in Japan (J-COSMOS). J. Gastroenterol..

[26] Yamakawa T., Ishigami K., Ohwada S., Kazama T., Hirayama D., Yoshii S., Yamano H.-O., Nakase H.A. (2023). Older Patient with Active Ulcerative Colitis and Coronavirus Disease 2019 (COVID-19) Pneumonia Successfully Treated with the Combination of Anti-TNFα Therapy and Azathioprine. Clin. J. Gastroenterol..

[27] Barazzoni R., Bischoff S.C., Breda J., Wickramasinghe K., Krznaric Z., Nitzan D., Pirlich M., Singer P. (2020). ESPEN Council ESPEN Expert Statements and Practical Guidance for Nutritional Management of Individuals with SARS-CoV-2 Infection. Clin. Nutr..

[28] Chen N., Zhou M., Dong X., Qu J., Gong F., Han Y., Qiu Y., Wang J., Liu Y., Wei Y. (2020). Epidemiological and Clinical Characteristics of 99 Cases of 2019 Novel Coronavirus Pneumonia in Wuhan, China: A Descriptive Study. Lancet.

[29] Ben Abdallah S., Mhalla Y., Trabelsi I., Sekma A., Youssef R., Bel Haj Ali K., Ben Soltane H., Yacoubi H., Msolli M.A., Stambouli N. (2023). Twice-Daily Oral Zinc in the Treatment of Patients With Coronavirus Disease 2019: A Randomized Double-Blind Controlled Trial. Clin. Infect. Dis..

[30] Thomas S., Patel D., Bittel B., Wolski K., Wang Q., Kumar A., Il’Giovine Z.J., Mehra R., McWilliams C., Nissen S.E. (2021). Effect of High-Dose Zinc and Ascorbic Acid Supplementation vs Usual Care on Symptom Length and Reduction Among Ambulatory Patients With SARS-CoV-2 Infection: The COVID A to Z Randomized Clinical Trial. JAMA Netw. Open.

[31] Wang B., Zhang L., Wang Y., Dai T., Qin Z., Zhou F., Zhang L. (2022). Alterations in Microbiota of Patients with COVID-19: Potential Mechanisms and Therapeutic Interventions. Signal Transduct. Target. Ther..

[32] Mak J.W.Y., Chan F.K.L., Ng S.C. (2020). Probiotics and COVID-19: One Size Does Not Fit All. Lancet Gastroenterol. Hepatol..

[33] Magro F., Rahier J.-F., Abreu C., MacMahon E., Hart A., van der Woude C.J., Gordon H., Adamina M., Viget N., Vavricka S. (2020). Inflammatory Bowel Disease Management During the COVID-19 Outbreak: The Ten Do’s and Don’ts from the ECCO-COVID Taskforce. J. Crohns Colitis.

[34] Steidler L., Hans W., Schotte L., Neirynck S., Obermeier F., Falk W., Fiers W., Remaut E. (2000). Treatment of Murine Colitis by Lactococcus Lactis Secreting Interleukin-10. Science.

[35] Trombetta A.C., Meroni M., Cutolo M. (2017). Steroids and Autoimmunity. Front. Horm. Res..

[36] Martineau A.R., Jolliffe D.A., Hooper R.L., Greenberg L., Aloia J.F., Bergman P., Dubnov-Raz G., Esposito S., Ganmaa D., Ginde A.A. (2017). Vitamin D Supplementation to Prevent Acute Respiratory Tract Infections: Systematic Review and Meta-Analysis of Individual Participant Data. Br. Med. J..

[37] Maghbooli Z., Sahraian M.A., Jamalimoghadamsiahkali S., Asadi A., Zarei A., Zendehdel A., Varzandi T., Mohammadnabi S., Alijani N., Karimi M. (2021). Treatment With 25-Hydroxyvitamin D3 (Calcifediol) Is Associated With a Reduction in the Blood Neutrophil-to-Lymphocyte Ratio Marker of Disease Severity in Hospitalized Patients With COVID-19: A Pilot Multicenter, Randomized, Placebo-Controlled, Double-Blinded Clinical Trial. Endocr. Pract..

[38] De Niet S., Trémège M., Coffiner M., Rousseau A.-F., Calmes D., Frix A.-N., Gester F., Delvaux M., Dive A.-F., Guglielmi E. (2022). Positive Effects of Vitamin D Supplementation in Patients Hospitalized for COVID-19: A Randomized, Double-Blind, Placebo-Controlled Trial. Nutrients.

[39] Kocot J., Luchowska-Kocot D., Kiełczykowska M., Musik I., Kurzepa J. (2017). Does Vitamin C Influence Neurodegenerative Diseases and Psychiatric Disorders?. Nutrients.

[40] Kaźmierczak-Barańska J., Boguszewska K., Adamus-Grabicka A., Karwowski B.T. (2020). Two Faces of Vitamin C-Antioxidative and Pro-Oxidative Agent. Nutrients.

[41] Liugan M., Carr A.C. (2019). Vitamin C and Neutrophil Function: Findings from Randomized Controlled Trials. Nutrients.

[42] Olczak-Pruc M., Swieczkowski D., Ladny J.R., Pruc M., Juarez-Vela R., Rafique Z., Peacock F.W., Szarpak L. (2022). Vitamin C Supplementation for the Treatment of COVID-19: A Systematic Review and Meta-Analysis. Nutrients.

[43] D’Ecclesiis O., Gavioli C., Martinoli C., Raimondi S., Chiocca S., Miccolo C., Bossi P., Cortinovis D., Chiaradonna F., Palorini R. (2022). Vitamin D and SARS-CoV2 Infection, Severity and Mortality: A Systematic Review and Meta-Analysis. PLoS ONE.

[44] Zhang L., Liu Y. (2020). Potential Interventions for Novel Coronavirus in China: A Systematic Review. J. Med. Virol..

[45] World Health Organisation Tobacco. https://www.who.int/news-room/fact-sheets/detail/tobacco.

[46] Farsalinos K., Barbouni A., Niaura R. (2020). Systematic Review of the Prevalence of Current Smoking among Hospitalized COVID-19 Patients in China: Could Nicotine Be a Therapeutic Option?. Intern. Emerg. Med..

[47] Changeux J.-P., Amoura Z., Rey F.A., Miyara M. (2020). A Nicotinic Hypothesis for Covid-19 with Preventive and Therapeutic Implications. Comptes Rendus Biol..

[48] Gallus S., Scala M., Possenti I., Jarach C.M., Clancy L., Fernandez E., Gorini G., Carreras G., Malevolti M.C., Commar A. (2023). The Role of Smoking in COVID-19 Progression: A Comprehensive Meta-Analysis. Eur. Respir. Rev..

[49] Monk R.L., Qureshi A.W., Richardson G.B., Heim D. (2023). UK Alcohol Consumption during the COVID-19 Pandemic: The Role of Drinking Motives, Employment and Subjective Mental Health. PLoS ONE.

[50] Sidor A., Rzymski P. (2020). Dietary Choices and Habits during COVID-19 Lockdown: Experience from Poland. Nutrients.

[51] Callinan S., Smit K., Mojica-Perez Y., D’Aquino S., Moore D., Kuntsche E. (2021). Shifts in Alcohol Consumption during the COVID-19 Pandemic: Early Indications from Australia. Addiction.

[52] Stanton R., To Q.G., Khalesi S., Williams S.L., Alley S.J., Thwaite T.L., Fenning A.S., Vandelanotte C. (2020). Depression, Anxiety and Stress during COVID-19: Associations with Changes in Physical Activity, Sleep, Tobacco and Alcohol Use in Australian Adults. Int. J. Environ. Res. Public Health.

[53] Vanderbruggen N., Matthys F., Van Laere S., Zeeuws D., Santermans L., Van den Ameele S., Crunelle C.L. (2020). Self-Reported Alcohol, Tobacco, and Cannabis Use during COVID-19 Lockdown Measures: Results from a Web-Based Survey. Eur. Addict. Res..

[54] Eşkazan T., Bakkaloğlu O.K., Durcan E., Kurt E.A., Önal U., Candan S., Tuncer M., Demirel Ö., Hatemi İ., Erzin Y. (2022). The Psychological Effects of COVID-19 Pandemic in Patients with Inflammatory Bowel Disease. Turk. J. Gastroenterol..

[55] Trindade I.A., Ferreira N.B. (2021). COVID-19 Pandemic’s Effects on Disease and Psychological Outcomes of People With Inflammatory Bowel Disease in Portugal: A Preliminary Research. Inflamm. Bowel. Dis..

[56] Taft T.H., Quinton S., Jedel S., Simons M., Mutlu E.A., Hanauer S.B. (2022). Posttraumatic Stress in Patients With Inflammatory Bowel Disease: Prevalence and Relationships to Patient-Reported Outcomes. Inflamm. Bowel. Dis..

[57] Nishida Y., Hosomi S., Fujimoto K., Nakata R., Itani S., Ohminami M., Nadatani Y., Fukunaga S., Otani K., Tanaka F. (2022). Effect of the Coronavirus Disease 2019 Lockdown on Lifestyle Factors in Japanese Patients with Inflammatory Bowel Disease. Intern. Med..

[58] Siegel C.A., Melmed G.Y., McGovern D.P., Rai V., Krammer F., Rubin D.T., Abreu M.T., Dubinsky M.C., International Organization for the Study of Inflammatory Bowel Disease (IOIBD), International Organization for the Study of Inflammatory Bowel Diseases (IOIBD) (2021). SARS-CoV-2 Vaccination for Patients with Inflammatory Bowel Diseases: Recommendations from an International Consensus Meeting. Gut.

[59] Pellegrino R., Pellino G., Selvaggi L., Selvaggi F., Federico A., Romano M., Gravina A.G. (2022). BNT162b2 mRNA COVID-19 Vaccine Is Safe in a Setting of Patients on Biologic Therapy with Inflammatory Bowel Diseases: A Monocentric Real-Life Study. Expert Rev. Clin. Pharmacol..

[60] Wellens J., Colombel J.-F., Satsangi J.J., Wong S.-Y. (2021). SARS-CoV-2 Vaccination in IBD: Past Lessons, Current Evidence, and Future Challenges. J. Crohn’s Colitis.

